# Multi-omics approaches reveal the mechanisms underlying the interaction between cyst fluid of *Echinococcus granulosus* and host immune cells

**DOI:** 10.3389/fimmu.2025.1598028

**Published:** 2025-08-20

**Authors:** Rongdong He, Ruofeng Yan, Yuanchun Shi, Aili Aierken, Xue Zhang, Hao Wen, Kalibixiati Aimulajiang

**Affiliations:** ^1^ State Key Laboratory of Pathogenesis, Prevention and Treatment of High Incidence Diseases in Central Asia, Clinical Medicine Institute, The First Affiliated Hospital of Xinjiang Medical University, Urumqi, China; ^2^ Department of Hepatobiliary and Hydatid Disease, Digestive and Vascular Surgery Center Therapy Center, The First Affiliated Hospital of Xinjiang Medical University, Urumqi, China; ^3^ Ministry of Education Joint International Research Laboratory of Animal Health and Food Safety, College of Veterinary Medicine, Nanjing Agricultural University, Nanjing, China; ^4^ Department of Rheumatology and Immunology, Friendship Hospital of Yili Kazakh Autonomous Prefecture, Yining, China

**Keywords:** *Echinococcus granulosus* cyst fluid, interleukin-9, splenic lymphocyte, metabolomics, transcriptomics

## Abstract

**Background:**

*Echinococcus granulosus* cyst fluid (EgCF) is a complex mixture of parasite’s containing a variety of antigens. Th9 cells are a newly reported subpopulation of Th cells whose primary function is to secrete IL-9 and exert biological effects. Research on whether antigens in the vesicle fluid can evade the host immune response by increasing IL-9 secretion is limited.

**Methods:**

The effects of EgCF on lymphocyte function in mice were evaluated using CCK-8 and flow cytometry for apoptosis. The effect of EgCF on CD4^+^IL-9^+^T cell differentiation was reflected by flow cytometry. The expression of TGF-β, IL-4, PU.1, IRF4 and IL-9 was detected by WB, qRT-PCR and ELISA under the influence of varying concentrations of EgCF. Analysis of differential metabolites and genes in mouse splenic lymphocytes was stimulated by EgCF using metabolomics and transcriptomics.

**Results:**

Different concentrations of EgCF stimulated lymphocytes, promoted cell proliferation and apoptosis, facilitated the differentiation of CD3^+^T cells and CD4^+^IL-9^+^T cells in splenic lymphocytes, and inhibited the differentiation of CD4^+^T cells. It regulated the host immune response by up-regulating Th9 cell-associated cytokines such as IL-4, TGF-β, IL-9 and related transcription factors PU.1 and IRF4. Metabolomic analysis identified 221 differential metabolites, 12 up-regulated and 11 down-regulated. These metabolites were primarily enriched in metabolic pathways such as beta-Alanine metabolism and Pyrimidine metabolism. Transcriptome analysis identified 16,694 differentially expressed genes, highlighting necroptosis and TGF-β signaling as top pathways, where Hgf and Myof were potential diagnostic markers.

**Conclusions:**

Metabolomics and transcriptomics analyses help identify potential candidate genes and provide diagnostic tools for future research and the discovery of new therapeutic targets. EgCF may regulates the host immune response by up-regulating Th9 cell-related cytokines such as IL-4, TGF-β and IL-9, along with related transcription factors PU.1 and IRF4. This provides a theoretical basis for understanding how *Echinococcus granulosus* modulates the host immune response and may offer new research avenues for immunoprophylaxis against *Echinococcus granulosus*.

## 1 Introduction

Cyst echinococcosis (CE) is a zoonotic parasitic disease caused by the infection of *Echinococcus granulosus*(*E. granulosus*). It has a worldwide distribution and is prevalent in western China, Central Asia, South America, Mediterranean countries, and East Africa (1, 2). *E. granulosus* parasitizes the liver, lungs and other tissues and organs of humans and animals for an extended period, causing varying degrees of harm to both humans and animals (3). The long-term survival of worms in humans or animals depends on immune evasion from the host (4). Currently, in addition to surgery, the main reliance is on chemical drugs to treat and prevent diseases. However, issues such as drug resistance and side effects have emerged. Immunoprophylaxis is considered one of the most promising and ideal preventive and control measures to control the prevalence of diseases (5). Therefore, an in-depth study of the pathogenesis and immune escape mechanisms of the disease is crucial theoretical guidance for the development of new vaccines.

*E. granulosus* exhibits a complex life cycle involving definitive hosts (canids) and intermediate hosts (herbivores/humans). The adult worm resides in the canine intestine, releasing eggs through feces. When ingested by intermediate hosts, the eggs hatch, releasing oncospheres that penetrate the intestinal wall and develop into metacestodes (hydatid cysts) in organs such as the liver or lungs over a period of 5 to 20 months. The cyst fluid (EgCF) analyzed in this study is derived from the metacestode stage, which secretes antigens to modulate the host’s immune response through several mechanisms: antigenic concealment facilitated by a carbohydrate-rich laminated layer, inhibition of macrophage activation by suppressing TRAF6 signaling, and promotion of Treg/Th2 responses that downregulate protective Th1 immunity. These mechanisms contribute to the long-term survival of the parasite (greater than 5 years in humans). However, the role of Th9 cells—a recently identified subset differentiated by the synergy of TGF-β and IL-4—in EgCF-mediated immune evasion remains unclear. EgCF is a complex mixed fluid containing not only the secretion of the worm and the surface shedding material of the worm but also some host proteins (6–11). Analysis of cyst fluid proteins revealed secreted proteins such as antigen B, peroxisomal proteins, and basement membrane-specific acetylheparin sulfate, among others. These highly expressed proteins may modulate the host immune system by influencing the cytokines or signaling pathways to facilitate immune escape. It has been found that *E. granulosus* antigen B affects human dendritic cell differentiation and induces dendritic cell differentiation towards Th2 cells (12). Thioredoxin peroxidase, a major component of peroxisomal proteins, regulates macrophage activation through the mTOR pathway, thereby promoting immune evasion by parasites (13). Glycomolecules in the EgCF can interfere with dendritic cell maturation and activation mediated by the TLR4 signaling pathway (11). EgCF suppresses inflammatory responses by inhibiting macrophage TRAF6 signaling (8). EgCF inhibits the expression of miR-19, thereby relieving its regulatory effect on the TGF-β receptor II (TβRII), which activates the TGF-β/Smad signaling pathway. This, in turn, drives the proliferation of hepatic stellate cells (HSCs) and collagen deposition (COL1A1/COL3A1), ultimately leading to fibrosis surrounding the hydatid cyst (14). The statement above implies that EgCF is involved in evading the host immune response. Helper T (Th) cells play a crucial role in the body’s immune response and are essential in combating parasitic infections. In malaria, Tregs may suppress protective immune responses but could also ameliorate disease, while in Leishmania infections, Tregs might maintain immunity to challenge infections by allowing the survival of residual parasite populations (15). However, the contribution of Th9 cells to immune evasion in *E. granulosus* is rarely reported.

Veldhoen (16) et al., 2008 and Dardalhon (17) et al., 2008 identified a novel subpopulation of effector CD4^+^T cells that predominantly secrete interleukin-9 (IL-9) and defined them as Th9 cells in 2008. When both TGF-β and IL-4 are present, their combined action can drive the differentiation of initial CD4^+^T cells toward Th9 cells (18). This finding reveals the important role of TGF-β and IL-4 in regulating Th9 cell differentiation in the immune system (19, 20). Th9 cells reduce parasite infection by producing IL-9 to increase mast cell numbers and drive basophils (21). Pang (22) demonstrated for the first time that the quantity of Th9 cells in the peripheral blood of patients with CE, along with the expression levels of IL-9 and PU.1, transcription factors associated with Th9 cells, were notably elevated compared to those of healthy controls. This suggests the involvement of Th9 cells in the immune response and the regulation of *E. granulosus* infection. Anuradha et al., 2013 found that circulating antigen-specific Th9 cells have been detected in patients with chronic *lymphatic filariasis* infection (23). In a mouse model of *Trichinella* sp*iralis* infection, IL-9 promotes helminth elimination by regulating intestinal muscle contraction, epithelial cell mucus production, and mast cell activity (24).

The application of metabolomics and transcriptomics provides a comprehensive understanding of biological processes and regulatory mechanisms in organisms (25, 26). The transcriptome reveals that pyronaridine has great effects on the spliceosome, MAPK pathway and ABC transporters, highlighting the mechanism by which pyronaridine kills *E. granulosus* protoscolices (27). Differential expression of antigen B, tegument antigen, and arginase-2 in sheep CE cysts obtained from liver and lung, as identified by transcriptome analysis (28). The identification of serum biomarkers is useful for early diagnosis by metabolomic analysis of differences in serum metabolic profiles of hepatic alveolar echinococcosis (AE), caused by *Echinococcus multilocularis*, and CE (29). Analysis of active and inactive liver cyst echinococcosis potential metabolic processes by metabolomics provides some clues for the diagnosis of CE (30).

In this study, we investigated the differential changes and genes analyzed through metabolomics and transcriptomics of mouse splenic lymphoid fine metabolites under varying mass concentrations of EgCF *in vitro*. The effects of varying mass concentrations of EgCF on the differentiated Th9 cell subpopulations of mouse splenic lymphocytes were investigated through *in vitro* incubation with mouse splenic lymphocytes. This study aims to enhance our understanding of host-parasite interactions.

## 2 Methods

### 2.1 Animals

Animals SPF grade BALB/c mice, 6–8 weeks old, female, weighing 18–20 g, were provided by the Medical Laboratory Animal Center of Xinjiang Medical University. The disposition of the animals during the experiments was in accordance with animal ethics standards (ethical approval number: IACUC20210301-01).

### 2.2 Preparation of the cyst fluid of *E. granulosus* and isolation of mouse lymphocyte

According to previous descriptions (31, 32), infected sheep livers containing *E. granulosus* larvae were obtained from a slaughterhouse in Urumqi. Under sterile conditions, cyst fluid was aspirated using a 16G needle to avoid contamination of host tissue. The fluid was immediately centrifuged at 4,000 × g for 15 minutes at 4°C to remove protoscoleces and debris. The supernatant was sequentially filtered through 0.8-μm and 0.22-μm PVDF membranes (Millipore). Protein concentration in the cyst fluid was determined using the BCA protein assay (Solarbio, Beijing, China), and aliquots were stored at -80°C for future use.

Female BALB/c mice were euthanized by decapitation. The spleens were aseptically removed, and the splenic tissues were ground using the frosted side of a slide^[17]^. The resulting mixture was filtered through a nylon mesh to collect splenic lymphocytes following the protocol of the mouse splenic lymphocyte isolation kit (Solarbio, Beijing, China). The isolated cells were counted and adjusted to a concentration of approximately 2×10^7^ cells/mL before being set aside.

### 2.3 Cell proliferation assay

Mouse splenic lymphocytes were cultured in RPMI-1640 medium (Gibco, Grand Island, NY, USA) supplemented with 10% fetal bovine serum, 100 μg/mL penicillin, and 100 U/mL streptomycin (HyClone, Logan, UT, USA) in a 5% CO_2_ atmosphere at 37°C in a cell culture incubator. To prepare the cell suspension, collect the cells and resuspend them in RPMI-1640 medium to a final concentration of 2×10^5^ cells per milliliter. Then, aliquot 5 μL of this cell suspension into each well of a 96-well plate, which will result in a seeding density of 1×10^4^ cells per well. Given that the concentration of cyst fluid protein is 3945 μg/mL, add varying volumes (0, 10.14, 20.28, 40.56 μL) of cyst fluid to the corresponding wells. Subsequently, supplement each well with RPMI-1640 medium to reach a total volume of 100 μL, thereby achieving final cyst fluid concentrations of (0, 100, 200, 400) μg/mL in the wells. Continue the cell culture for an additional 24 hours under standard conditions. Afterward, 10 µL of CCK-8 reagent (Beyotime, Jiangsu, China) was added, with cell-free wells serving as negative controls, and the incubation was continued for an additional 4 hours. Each group includes three replicate wells, and the experiment was repeated three times. The optical density value (OD value) was detected at 450 nm using an enzyme labeling instrument. The cell proliferation rate was calculated using the formula: Proliferation rate (%) = [(OD of experimental wells - OD of negative control wells)/(OD of control wells - OD of negative control wells)]×100%.

### 2.4 Annexin V-­FITC/propidium iodide staining assay

Splenic lymphocytes were collected and 1×10^6^ cells were dispensed per well in 24-well plates, with 3 replicate wells in each group. Subsequently, they were treated with different concentrations (0, 100, 200, 400 μg/mL) of EgCF and then incubated for 24h. Apoptosis was detected using the steps outlined in the Annexin V-FITC Apoptosis Assay Kit (BD Biosciences, San Jose, CA, USA). Apoptotic cells were classified as: early apoptotic (Annexin V^+^/PI^−^), late apoptotic (Annexin V^+^/PI^+^), and total apoptosis rate was the sum of both populations. The cell apoptosis rate (%) was calculated using the formula: total apoptosis rate (%) = early apoptosis rate (%) + late apoptosis rate (%).

### 2.5 Flow cytometry analysis

Splenic lymphocytes were collected and 2×10^5^ cells were dispensed per well in 24-well plates, with 3 replicate wells in each group. Subsequently, different concentrations (0, 100, 200, 400 μg/mL) of EgCF were added to the wells for incubation for 24 hours. Cells were resuspended in a stimulant containing Leukocyte Activation Cocktail (Cat#554656, 2 µL of cocktail for every 1 mL of cell culture, BD Biosciences, Franklin Lakes, NJ, USA) culture medium. After 4 hours of stimulation culture, the cells were transferred to 1.5 mL centrifuge tubes. Subsequently, the cells were resuspended in a blocking solution containing Anti-Mouse CD16/32 ((Fc Block, Clone 93, Cat# 101302,BioLegend, San Diego, CA, USA) diluted 1:100 in Stain Buffer (PBS with 0.1% BSA and 0.09% sodium azide) at 4°C for 20 minutes. Labeled antibodies CD3(Clone 17A2, Cat# 100204,BioLegend, diluted 1:200,San Diego, CA, USA) and CD4 (Clone GK1.5, Cat# 100433, diluted 1:200,BioLegend, San Diego, CA, USA) were added separately. The cell membrane was disrupted, and the cell suspension was incubated with the antibody IL-9 (Clone RM9A4, Cat# 514111, diluted 1:50,BioLegend, San Diego, CA, USA) for 30 minutes at 4°C in the dark. Subsequently, the cells were resuspended in Stain Buffer. All samples were analyzed using an LSRFortessa flow cytometer (BD Immunocytometry Systems, San Jose, CA, USA), and the data were processed with FlowJo software (version V10; TreeStar Inc., Ashland, OR, USA).

### 2.6 Cytokine detection

Splenic lymphocytes were collected and 1×10^6^ cells were dispensed per well in 24-well plates, with three replicate wells in each group. Subsequently, different concentrations (0, 100, 200, 400 μg/mL) of EgCF were added to continue the incubation for 24 hours. The cells were then collected and inoculated in 24-well plates at a density of 1×10^6^ cells/well, with three replicate wells in each group. The supernatant was collected, and the levels of IL-4, TGF-β and IL-9 cytokines were determined using an ELISA kit following the manufacturer’s instructions (Nanjing Lapuda Biotechnology Co., Ltd, Nanjing, China).

### 2.7 Real-time quantitative PCR

Splenic lymphocytes were collected and RNA was extracted using a total cellular RNA isolation kit (Foregene, Chengdu, China) from 2×10^6^ cells per experimental group after 24-hour stimulation with EgCF (0, 100, 200, 400 μg/mL). The RNA was then reverse transcribed using the HiScript III RT SuperMix kit (Vazyme, Nanjing, China) following the manufacturer’s instructions. qRT-PCR was performed using ChamQ SYBR qPCR Master Mix (Vazyme) and run on a qRT-PCR instrument (iQ 5 Bio-Rad, Hercules, CA, USA). The target genes analyzed included IL-4,TGF-β,IL-9,PU.1, and IRF4. The reaction conditions were as follows: stage 1, 95°C for 30 s; stage 2, 40 cycles of 95°C for 5 s and 60°C for 30 s; and stage 3, melting at 95°C for 10 s, 65°C for 5 s, 95°C for 1 s melting curve analysis. The relative expression of the target genes was determined using the comparative quantification cycle (Cq) normalized against the housekeeping gene (β-actin) with the 2^-ΔΔCq^ method. All primers utilized in this analysis were synthesized by Anhui General Biological Co., as detailed in **Table 1**.

**Table 1:** Primer sequences for quantitative real time PCR.

Gene	Forward primer	Reverse primer
IL-4	CTCACTCTCTGTGGTGTTCT	CTCCCTCACAATTTCCATCC
IL-9	GACATACATCCTTGCCTCTGTT	CGGTGTGGTACAATCATCAGT
IRF4	GCCCAACAAGCTAGAAAGAG	CTGGAAACTCCTCACCAAAG
PU.1	GACAGGCGAGGTGAAGAAAG	GGCGACGGGTTAATGCTATG
TGF-β	TGACGTCACTGGAGTTGTACGG	GGTTCATGTCATGGATGGTGC
β-actin	CCGTAAAGACCTCTATGCCAAC	GGGTGTAAAACGCAGCTCAGTA

### 2.8 Western blot analysis

Splenic lymphocytes (2×10^6^ cells per sample) were collected after 24-hour stimulation with EgCF (0, 100, 200, 400 μg/mL) and lysed in RIPA buffer containing 50 mM Tris-HCl (pH 7.4), 150 mM NaCl, 1% NP-40, 0.5% sodium deoxycholate, 0.1% SDS, supplemented with phosphatase and protease inhibitor cocktail (EMD Millipore). Protein sample concentration was determined by BCA Protein Quantification kit (Solarbio, Beijing, China). Twenty micrograms of protein were separated by 10% SDS-PAGE, transferred to polyvinylidene fluoride (PVDF) membranes (Millipore Corp., MA, USA), and incubated with rabbit anti-IFR4 (1:1000), anti-PU.1 (1:1000), anti-IL-9 (1:1000), anti-PCNA (1:1000), anti-Cyclin D1 (1:1000), or anti-GAPDH (1:10000) diluted in TBS-T (Tris-buffered saline with 0.1% Tween 20). The membrane was blocked with 5% skim milk in TBS-T for 2h and then the antibody was added to the shaker at 4°C for 12h. The membranes were then incubated with alkaline phosphatase-conjugated anti-rabbit IgG antibodies (1:5000; Cell Signaling Technology, Danvers, MA, USA) in TBS-T for 2 hours. The immune complexes were visualized with the ECL substrate (Biosharp BL520A) on a gel imager (Bio-Rad Laboratories, Inc.,USA).

### 2.9 Metabolome detection and analysis

Splenic lymphocytes (2×10^6^ cells per sample) after 24-hour stimulation with EgCF (0, 100, 200, 400 μg/mL) were collected for qualitative and quantitative analysis of metabolites using liquid chromatography tandem mass spectrometry (LC-MS/MS) with technical support from Suzhou Panomic Biomedical Technology Co (33). Samples were analyzed on a Q Exactive HF-X system (Thermo Fisher) with HILIC chromatography (Waters XBridge BEH Amide column). Metabolite separation used gradient elution: mobile phase A (5 mM ammonium acetate in water, pH 9.0), B (acetonitrile). Full scan range: m/z 70-1050, resolution 70,000.The metabolites were identified using accurate mass and MS/MS data, which were matched with databases such as HMDB (http://www.hmdb.ca), MassBank (http://www.massbank.jp/), KEGG (https://www.genome.jp/kegg/), LipidMaps (http://www.lipidmaps.org), mzCloud (https://www.mzcloud.org), and the metabolite database built by Panomix Biomedical Tech Co., Ltd. (Suzhou, China). Variable importance in the projection (VIP) describes the overall contribution of each variable to the model, and the threshold is usually set at VIP > 1. Differential metabolites were identified using |log2FoldChange| ≥ 1 and P < 0.05 (34).

### 2.10 Transcriptomics detection and analysis

Splenic lymphocytes (2×10^6^ cells per sample) after 24-hour stimulation with EgCF (0, 100, 200, 400 μg/mL) were collected for transcriptome analysis. RNA-seq was performed on Illumina NovaSeq 6000 (150 bp paired-end). Library preparation used NEBNext Ultra II RNA Kit. Differential gene expression analysis was conducted with DESeq2.Differentially expressed genes were identified based on the criteria of |log2FoldChange| ≥ 1 and False Discovery Rate (FDR) <0.05. Genes were considered up-regulated when log2FoldChange > 1 and down-regulated when log2FoldChange < 1. GO and KEGG enrichment analysis of differential genes was conducted using the GO database (http://www.geneontology.org/) and the KEGG database (35) (Kyoto Encyclopedia of Genes and Genomes). GO and KEGG pathway analysis was conducted to identify significantly enriched pathways among differentially expressed genes, using a q-value (Benjamini-Hochberg corrected) threshold of < 0.05 for screening.

### 2.11 Combined transcriptomics and metabolomics analysis

Differential metabolites and differentially expressed genes were annotated in the KEGG database. Two-way orthogonal partial least squares (O2PLS) analysis was conducted to evaluate sample quality and histological correlation. Subsequently, KEGG enrichment analysis was carried out to identify jointly significant enrichment pathways. This was followed by the integration of clustering analysis results from transcriptome and metabolome to investigate the regulatory mechanisms between genes and metabolites.

### 2.12 Statistical analysis

The results are presented as the mean ± standard error of the mean (SEM) and were analyzed using GraphPad Prism 8.0.2 software (GraphPad Software, Inc., USA). The statistical significance was assessed by one-way ANOVA with Tukey’s multiple comparison test, and Student’s t-test was used for the comparisons of only two groups. Differences were considered significant at *p < 0.05, **p < 0.01, ***p < 0.001, ****p < 0.0001.

## 3 Results

### 3.1 EgCF promotes the proliferation and apoptosis of mouse splenic lymphocytes

**Figure 1:**
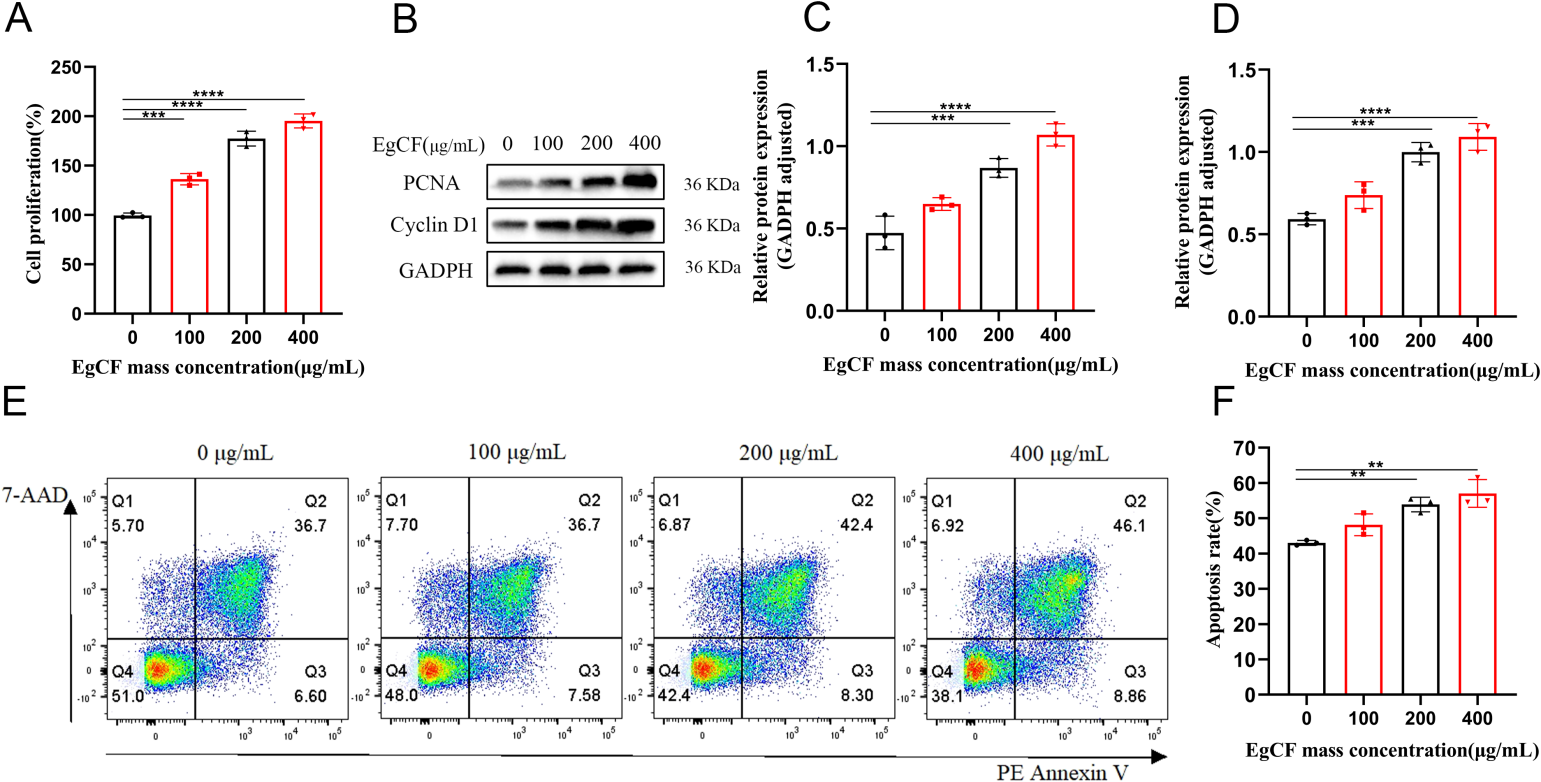
Effects of EgCF on proliferation and apoptosis of mouse splenic lymphocytes. **(A)** Cell proliferation detected by CCK-8; **(B)** PCNA and PCNA Western blot graph; **(C)** PCNA protein level detected by Western blot; **(D)** Cyclin D1 protein level detected by Western blot; **(E)** apoptosis rate of lymphocytes in each group **(F)** Effect of EgCF on apoptosis of mouse splenic lymphocytes. P values were showed as: **p < 0.01, ***p < 0.001, ****p < 0.0001.

Mouse splenic lymphocytes were co-incubated with various concentrations (0, 100, 200, 400 μg/mL) of EgCF for 24 h *in vitro*, and cell proliferation was determined by measuring the absorbance with the CCK-8 cell proliferation assay. As the mass concentration of EgCF increased, cell proliferative activity was elevated in a dose-dependent manner compared to the control group (**Figure 1A**, p < 0.001). To confirm the proliferative effect at the molecular level, we examined the expression of proliferation markers PCNA (a DNA replication processivity factor) and Cyclin D1 (a key regulator of G1/S phase transition). The expression of PCNA was detected using a protein blotting assay, and it increased with the rise in EgCF concentration (**Figures 1B, C**, *p* < 0.001). Cyclin D1 expression was detected using a protein blotting assay, and its levels increased with the rise in EgCF concentration (**Figures 1B, D**, *p* < 0.001). It is suggested that EgCF has a proliferation-promoting effect on mouse splenic lymphocytes. In addition, we investigated the effect of EgCF on apoptosis in mouse splenic lymphocytes. Our data showed that the total apoptosis rate increased with the rise in the mass concentration of EgCF. Although statistically significant, this increase was modest (approximately 10% at 400 μg/mL) compared to the robust proliferation induction (over 2-fold increase). The increase was in the 200 μg/mL and 400 μg/mL groups compared to the control group (**Figures 1E, F**, *p* < 0.01). This indicates that while EgCF primarily promotes lymphocyte proliferation, it may also induce apoptosis in a subset of cells at higher concentrations.

### 3.2 EgCF promotes the differentiation of CD4^+^IL-9^+^T cells in splenic lymphocyte

**Figure 2:**
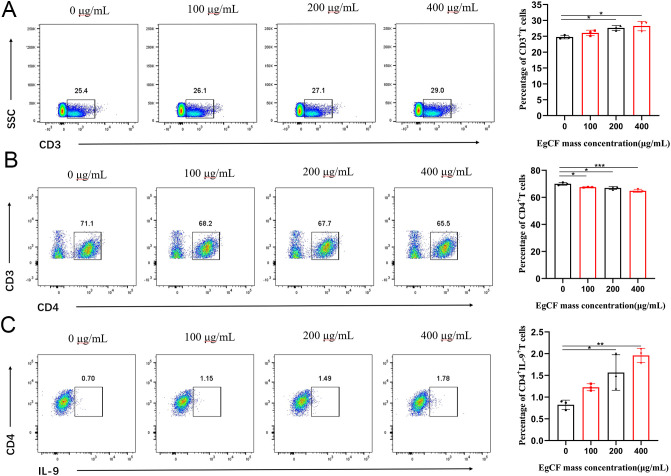
Effects of different concentrations of EgCF on splenic lymphocyte subpopulations in mice. Results of cell subpopulations detected by flow cytometry after incubation of EgCF and splenic lymphocytes, **(A)** CD3^+^T cells, **(B)** CD4^+^T cells, **(C)** CD4^+^IL-9^+^T cells. P values were showed as: *p < 0.05, **p < 0.01, ***p < 0.001.

**Figure 3:**
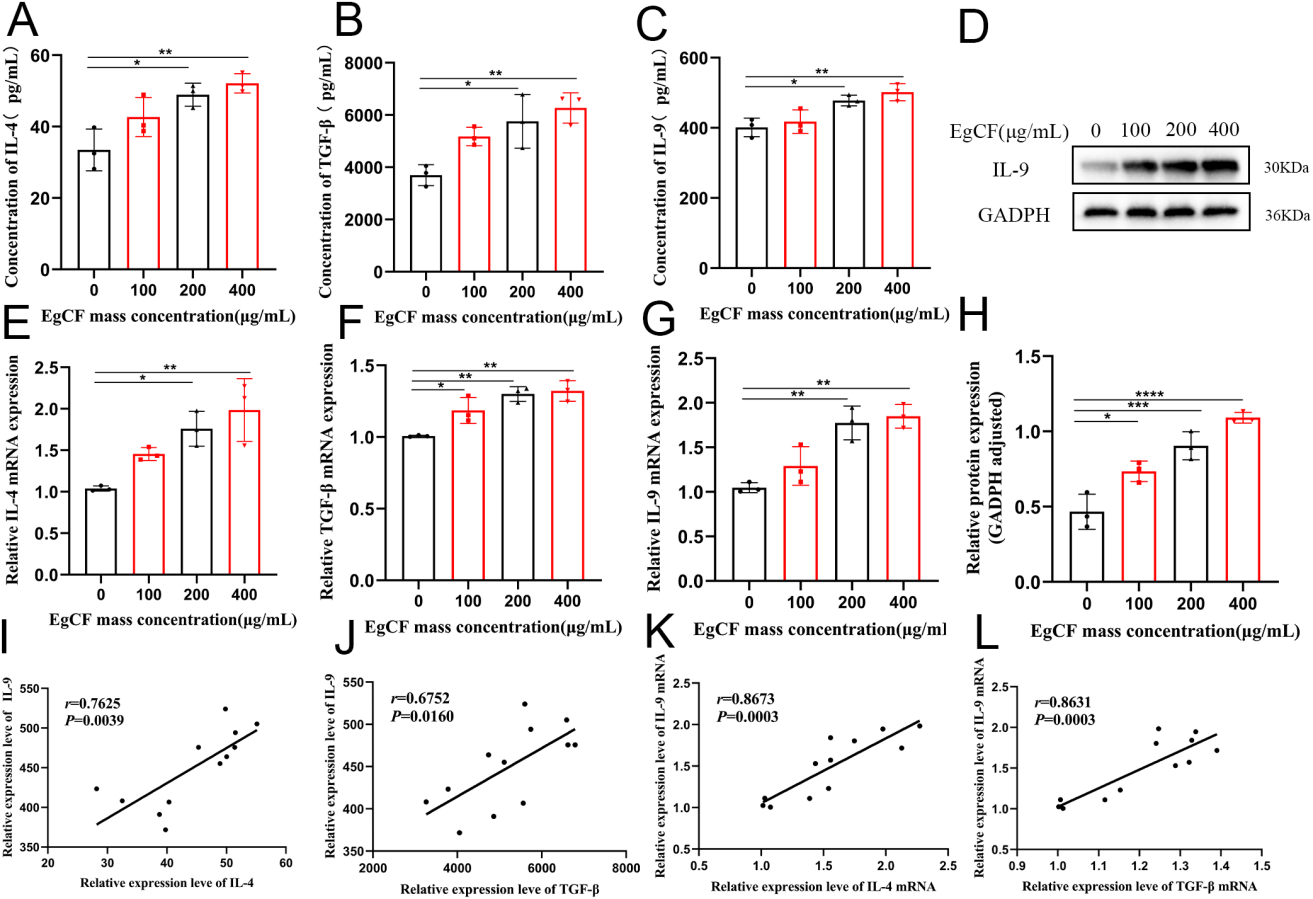
Effect of EgCF on the expression of IL-4, TGF-β and IL-9 by splenic lymphocytes. **(A)** ELISA for IL-4 expression; **(B)** ELISA for TGF-β expression; **(C)** ELISA for IL-9 expression; **(D)** IL-9 Western Blot graph; **(E)** qRT-PCR for IL-4 mRNA level; **(F)** qRT-PCR for TGF-β level; **(G)** qRT-PCR for IL-9 levels; **(H)** Western Blot for IL-9 protein levels; **(I)** ELISA for correlation analysis between IL-9 expression levels and IL-4 expression levels; **(J)** ELISA for correlation analysis between IL-9 expression levels and TGF-β expression levels; **(K)** qRT-PCR for correlation analysis between IL-9 mRNA expression levels and IL-4 mRNA expression level correlation analysis; **(L)** qRT-PCR detection of IL-9 mRNA expression level and TGF-β mRNA expression level correlation analysis. P values were showed as: *p < 0.05, **p < 0.01, ***p < 0.001, ****p < 0.0001.

The cells collected after stimulation with EgCF were analyzed using flow cytometry. The proportion of CD3^+^T cells expressed in splenic lymphocytes increased gradually with the increase of EgCF mass concentration. It was significantly higher in the 200 μg/mL group and 400 μg/mL group compared with the control group (**Figure 2A**, p < 0.05). The proportion of CD4^+^T cells expressed in splenic lymphocytes gradually decreased with the increase of EgCF mass concentration. It was significantly lower in the 100 μg/mL, 200 μg/mL, and 400 μg/mL groups compared with the control group (**Figure 2B**, p < 0.05). The values of CD4^+^IL-9^+^T cells in splenic lymphocytes increased gradually with the increase in mass concentration of EgCF and were significantly higher in the 200 μg/mL group and 400 μg/mL group compared with the control group (**Figure 2C**, p < 0.05). While this study focused on Th9 differentiation, the observed elevated IL-4 levels (**Figures 3A, E**) suggest concurrent Th2 activation, consistent with the known role of IL-4 in Th9 polarization. The reduction in CD4^+^T cells (**Figure 2B**) may reflect subset-specific differentiation rather than overall depletion, as CD3^+^T cells increased (**Figure 2A**). Notably, EgCF-induced apoptosis (**Figure 1E**) was modest compared to proliferation (**Figure 1A**), implying that lymphocyte expansion is driven by specific Th subsets (e.g., Th9/Th2) rather than global T cell activation.

### 3.3 EgCF promotes the expression of IL-4, TGF-β and IL-9 in splenic lymphocytes

It has been shown that CD4^+^T cells can differentiate into predominantly IL-9-secreting Th9 cells under IL-4 and TGF-β co-stimulation (18). Cells were collected after co-incubated to detect the expression of IL-4, TGF-β and IL-9 mRNA by qRT-PCR. The expression ratio of IL-4 mRNA increased gradually with the increase of EgCF mass concentration and was significantly higher in the 200 μg/mL group and 400 μg/mL group compared with the control group (**Figure 3E**, p < 0.05). The expression ratio of TGF-β mRNA increased gradually with the increase of EgCF mass concentration and was significantly higher in the 100 μg/mL group, 200 μg/mL group, and 400 μg/mL group compared with the control group (**Figure 3F**, p < 0.05). The expression of IL-9 mRNA gradually increased with the increase of EgCF mass concentration and was significantly higher in the 200 μg/mL group and 400 μg/mL group compared with the control group (**Figure 3G**, p < 0.01). Correlation analysis of the qRT-PCR results showed a positive correlation between IL-9 mRNA expression level and IL-4 mRNA expression level (**Figure 3K**, r = 0.8673, *p* = 0.0003), as well as a positive correlation between IL-9 mRNA expression level and TGF-β mRNA expression level (**Figure 3L**, r = 0.8631, *p* = 0.0003).

Cells were collected after co-incubated to detect IL-9 expression by Western blot. The results showed that the expression ratio of IL-9 increased gradually with the increase of EgCF mass concentration, and the groups with 100 μg/mL, 200 μg/mL and 400 μg/mL were significantly higher compared to the control group (**Figures 3D, H**, *p* < 0.05).

Supernatants of the cells after co-incubated were collected to detect the expression of IL-4, TGF-β and IL-9 in the supernatants using ELISA. The expression of IL-4 in the co-incubated supernatants increased proportionally with the increase of the mass concentration of EgCF and was significantly higher in the 200 μg/mL and 400 μg/mL groups compared to the control group (**Figure 3A**, p < 0.05). The expression of TGF-β in the co-incubated supernatants gradually increased in proportion to the mass concentration of EgCF, and was significantly higher in the 200 μg/mL group and 400 μg/mL group compared with the control group (**Figure 3B** ,p < 0.05). The expression of IL-9 in the co-incubated supernatants exhibited a gradual increase in value with the rising mass concentration of EgCF. It was notably higher in the 200 μg/mL and 400 μg/mL groups compared to the control group (**Figure 3C**, p < 0.05). The correlation analysis of the ELISA results was performed, revealing a positive correlation between the expression levels of IL-9 and IL-4 (**Figure 3I**, r = 0.7625, *p* = 0.0039), as well as a positive correlation between the expression levels of IL-9 and TGF-β (**Figure 3J**, r = 0.6752, *p* = 0.0160).

### 3.4 EgCF promotes the expression of PU.1 and IRF4 in splenic lymphocytes

**Figure 4:**
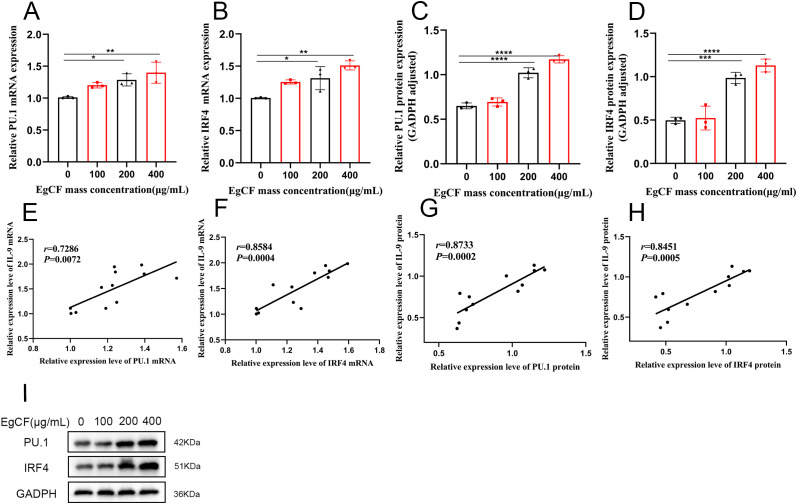
Effect of EgCF on the expression of PU.1 and IRF4 in splenic lymphocytes. **(A)** qRT-PCR for PU.1 mRNA level; **(B)** qRT-PCR for IRF4 mRNA level; **(C)** Western Blot for PU.1 protein level; **(D)** Western Blot for IRF4 protein level; **(E)** qRT-PCR for IL-9 mRNA expression level and PU.1 mRNA expression level correlation analysis; **(F)** qRT-PCR detection of IL-9 mRNA expression level and IRF4 mRNA expression level correlation analysis; **(G)** Western Blot detection of IL-9 protein level and PU.1 protein level correlation analysis; **(H)** Western Blot detection of IL-9 protein level and PU.1 protein level correlation analysis; **(I)** PU.1 and IRF4 Western Blot graph. P values were showed as: *p < 0.05, **p < 0.01, ***p < 0.001, ****p < 0.0001.

It was shown that PU.1 and IRF4 are key transcription factors for Th9 cell differentiation (36, 37). Cells were collected after co-incubated to detect the expression of PU.1 and IRF4 mRNA using qRT-PCR. The mRNA expression ratio increased gradually with the rise in the mass concentration of EgCF, being significantly higher in the 200 μg/mL and 400 μg/mL groups compared to the control (*p* < 0.05, **Figure 4A**). The expression ratio of IRF4 mRNA increased gradually with the increase of EgCF mass concentration and was significantly higher in the 200 μg/mL group and 400 μg/mL group compared with the control group (**Figure 4B**, p < 0.05). Correlation analysis of qRT-PCR results revealed a positive correlation between IL-9 mRNA expression level and PU.1 mRNA expression level (**Figure 4E**, r = 0.7286, *p* = 0.0072), as well as a positive correlation between IL-9 mRNA expression level and IRF4 mRNA expression level (**Figure 4F**, r = 0.8584, *p* = 0.0004).

Cells were collected after co-incubated to detect the expression of PU.1 and IRF4 by Western blot. The expression ratio of PU.1 increased gradually with the increase of EgCF mass concentration and was significantly higher in the 200 μg/mL group and 400 μg/mL group compared with the control group (**Figures 4C, I**, *p* < 0.001). The ratio of IRF4 expression increased gradually with the rise in EgCF mass concentration, and was significantly higher in the 200 μg/mL group and 400 μg/mL group compared to the control group (**Figures 4D, I**, *p* < 0.001). Correlation analysis of Western blot results was conducted, revealing a positive correlation between IL-9 expression level and PU.1 expression level (**Figure 4G**, r = 0.7286, *p* = 0.0072), as well as a positive correlation between IL-9 expression level and IRF4 expression level (**Figure 4H**, r = 0.8584, *p* = 0.0004).

### 3.5 Analysis of metabolite differences after co-incubated of EgCF with mouse splenic lymphocytes

**Figure 5:**
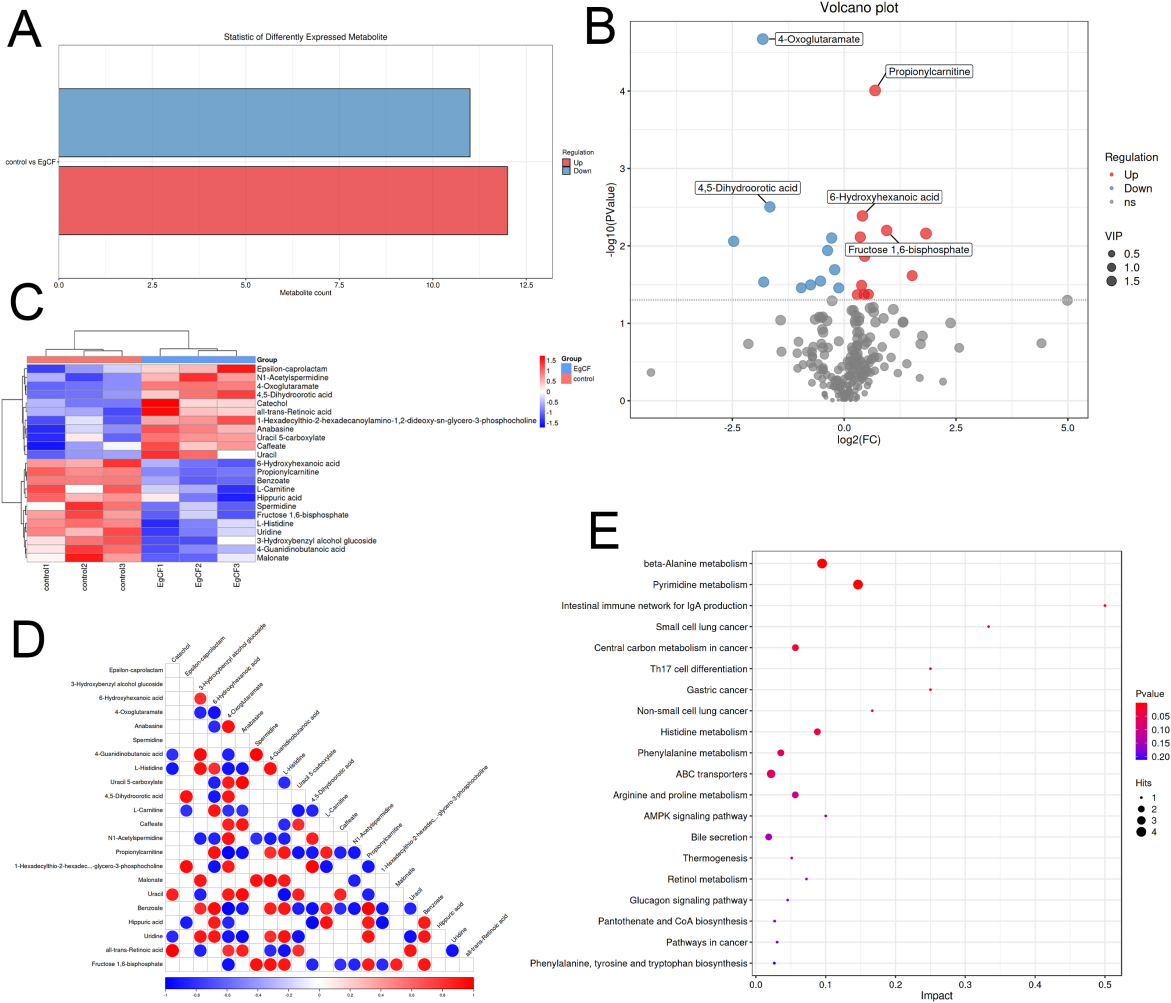
Metabolomic analysis of EgCF after co-incubated with mouse splenic lymphocytes. **(A)** Graph of differential metabolite statistics with colors where red indicates up-regulated metabolites and green indicates down-regulated metabolites. **(B)** Differential metabolite volcano plot, with red dots representing upregulated metabolites, blue dots representing downregulated metabolites, and gray dots indicating metabolites that did not meet the criteria for differential screening. **(C)** Heatmap of differential metabolite clustering, with red indicating high expression levels and blue indicating low expression levels. **(D)** Differential metabolite association heatmap with colors representing correlation: red indicates positive correlation, blue indicates negative correlation, and the darker the color the stronger the correlation. **(E)** Bubble diagram of metabolic pathway impact factors. Dot size represents the number of metabolites associated with the pathway, while color indicates the P-value. Redder colors signify smaller P-values, while bluer colors indicate larger P-values.

Metabolomic analysis identified 221 differential metabolites (VIP > 1, |log_2_FC| ≥ 1, p < 0.05), including 12 significantly up-regulated metabolites (e.g., spermidine, benzoate) and 11 down-regulated metabolites (e.g., all-trans-retinoic acid, catechol) (**Table 2**). The overall distribution of these metabolites was visualized in a volcano plot (**Figures 5A, B**), while hierarchical clustering revealed distinct expression patterns across samples (**Figure 5C**). KEGG enrichment analysis demonstrated predominant enrichment in β-Alanine metabolism, Pyrimidine metabolism, and Glyoxylate and dicarboxylate metabolism. Notably, permidine was markedly up-regulated in the β-Alanine metabolism pathway. This polyamine inhibits macrophage activation by suppressing TRAF6 signaling, aligning with EgCF’s immunosuppressive role. Conversely, all-trans-retinoic acid may promote myeloid-derived suppressor cell (MDSC) differentiation, suggesting EgCF reprograms immune cell fates. Benzoate, a TLR4 pathway modulator, potentially regulates Th9-associated cytokines (e.g., IL-4/TGF-β). Within the β-Alanine metabolism pathway (containing 32 metabolites), 4-oxoglutaramate and propionylcarnitine showed the strongest correlation with EgCF concentration (**Figure 5D**), further implicating metabolic rewiring in immune evasion mechanisms.

**Table 2:** Key differential metabolites of mouse spleen lymphocytes.

Different metabolites	mz	rt	P-value	FC	Variation trend
Benzoate	121.02	37.2	6.92×10^−3^	3.56	↑
Spermidine	146.17	38.4	2.42×10^−2^	2.87	↑
Fructose 1,6-bisphosphate	338.99	37.4	6.31×10^−3^	1.94	↑
Propionylcarnitine	218.14	83.7	9.82×10^−5^	1.62	↑
Uridine	243.06	62.3	1.13×10^−2^	1.5	↑
Hippuric acid	178.05	79.7	4.21×10^−2^	1.45	↑
4-Guanidinobutanoic acid	146.09	53.2	1.35×10^−2^	1.37	↑
Malonate	102.96	87	4.25×10^−2^	1.36	↑
6-Hydroxyhexanoic acid	132.08	49.1	4.10×10^−3^	1.33	↑
3-Hydroxybenzyl alcohol glucoside	124.09	688.2	3.23×10^−2^	1.31	↑
L-Histidine	156.08	42.1	7.68×10^−3^	1.28	↑
L-Carnitine	162.11	47.9	4.27×10^−2^	1.22	↑
Caffeate	163.04	386.6	3.49×10^−2^	0.92	↓
Uracil	111.02	58.5	2.02×10^−2^	0.87	↓
N1-Acetylspermidine	188.18	41.3	7.91×10^−3^	0.83	↓
Anabasine	144.98	688.4	1.14×10^−2^	0.77	↓
all-trans-Retinoic acid	299.20	555.1	2.84×10^−2^	0.69	↓
Uracil 5-carboxylate	156.97	34.7	3.18×10^−2^	0.59	↓
Catechol	111.02	36.8	3.48×10^−2^	0.52	↓
4,5-Dihydroorotic acid	158.96	252.2	3.13×10^−3^	0.32	↓
Epsilon-caprolactam	114.09	579.4	2.92×10^−2^	0.29	↓
4-Oxoglutaramate	145.05	192.9	2.13×10^−5^	0.28	↓
1-Hexadecylthio-2-hexadecanoylamino-1,2-dideoxy-sn-glycero-3-phosphocholine	718.58	570.4	8.71×10^−3^	0.18	↓

“↑” and “↓” mean that the compound is upregulated and downregulated.

### 3.6 Analysis of differentially expressed genes after co-incubated of EgCF with mouse splenic lymphocytes

**Figure 6:**
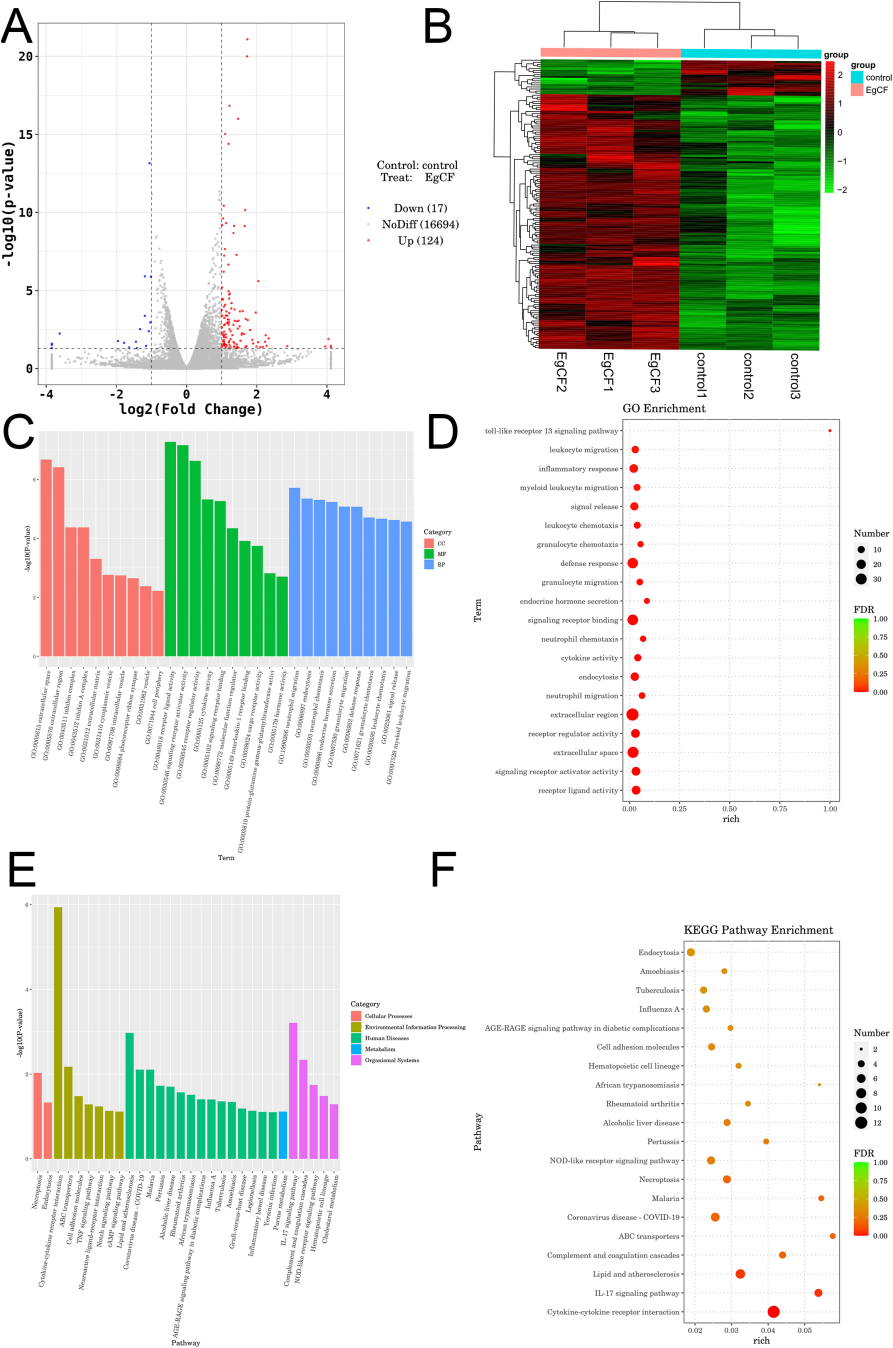
Transcriptomic analysis of EgCF after co-incubated with mouse splenic lymphocytes. **(A)** Volcano plot displaying differentially expressed genes. Red dots represent up-regulated genes in the group, blue dots represent down-regulated genes in the group, and gray dots represent genes that are not significantly differentially expressed. **(B)** Heatmap illustrating clustering of differentially expressed genes, with red indicating high expression levels and green indicating low expression levels. **(C)** Bubble diagram illustrating the Gene Ontology (GO) enrichment analysis of differentially expressed genes. Dot size represents the number of metabolites associated with the pathway, while color indicates the P-value; red denotes smaller P-values, and green indicates larger P-values. **(D)** Histogram showing the results of Gene Ontology enrichment analysis of differentially expressed genes. **(E)** Bubble diagram illustrating KEGG enrichment analysis of differentially expressed genes. Dot size *Echinococcus granulosus* represents the number of corresponding metabolites in the pathway, while color indicates P-value; red denotes smaller P-values and green indicates larger P-values. **(F)** Histogram of enrichment results for the differentially expressed gene KEGG Pathway.

Transcriptomic analysis identified 141 differentially expressed genes (DEGs) from an initial screening of 16,694 genes, comprising 124 upregulated genes (e.g., hepatocyte growth factor *Hgf*; myoferlin *Myof*, and 17 downregulated genes (e.g., natural cytotoxicity receptor *Ncr1*, low-density lipoprotein receptor *Ldlr*) (**Table 3**). Visualization included a volcano plot highlighting DEG distribution (**Figure 6A**) and a hierarchical clustering heatmap depicting expression patterns, with red indicating high expression and blue indicating low expression (**Figure 6B**). GO enrichment analysis revealed significant terms across categories (**Figures 6C, D**, **Table 3**), including neutrophil migration in Biological Process, receptor ligand activity in Molecular Function, and extracellular space in Cellular Component. KEGG pathway analysis identified key enriched pathways, including necroptosis (18 DEGs including RIPK1 and MLKL), TGF-β signaling, and IL-17 signaling, spanning cellular processes, environmental information processing, human diseases, metabolism, and organismal systems (**Figures 6E, F**). Critically, neutrophil migration (top BP term) mechanistically links to Th9 cells: IL-9 secretion induces epithelial production of CXCL1/CXCL2 chemokines, recruiting neutrophils to form granulomas around parasites. This process may facilitate *E. granulosus* immune evasion by containing infection while promoting chronic inflammation.

**Table 3:** GO enrichment analysis of the DEGs of mouse spleen lymphocytes.

Category	GO term ID	GO term description	DEG	P-value
Cellular component	GO:0005615	extracellular space	31	2.10×10^−7^
	GO:0005576	extracellular region	39	3.86×10^−7^
	GO:0043511	inhibin complex	2	4.26×10^−5^
	GO:0043512	inhibin A complex	2	4.34×10^−5^
	GO:0031012	extracellular matrix	11	4.95×10^−4^
	GO:0031410	cytoplasmic vesicle	18	1.73×10^−3^
	GO:0097708	intracellular vesicle	25	1.81×10^−3^
	GO:0098684	photoreceptor ribbon synapse	2	2.25×10^−3^
	GO:0031982	vesicle	25	4.22×10^−3^
	GO:0071944	cell periphery	57	6.07×10^−3^
Molecular function	GO:0048018	receptor ligand activity	17	5.37×10^−8^
	GO:0030546	signaling receptor activator activity	17	6.89×10^−8^
	GO:0030545	receptor regulator activity	17	2.34×10^−7^
	GO:0005125	cytokine activity	10	4.77×10^−6^
	GO:0005102	signaling receptor binding	28	5.42×10^−6^
	GO:0098772	molecular function regulator	28	4.56×10^−5^
	GO:0005149	interleukin-1 receptor binding	3	1.21×10^−4^
	GO:0038024	cargo receptor activity	5	1.79×10^−4^
	GO:0005179	hormone activity	5	1.98×10^−3^
	GO:0005545	1-phosphatidylinositol binding	2	3.23×10^−3^
Biological process	GO:1990266	neutrophil migration	8	1.91×10^−6^
	GO:0006897	endocytosis	15	4.45×10^−6^
	GO:0030593	neutrophil chemotaxis	7	4.93×10^−6^
	GO:0060986	endocrine hormone secretion	6	5.77×10^−6^
	GO:0097530	granulocyte migration	8	8.35×10^−6^
	GO:0006952	defense response	27	8.46×10^−6^
	GO:0071621	granulocyte chemotaxis	7	1.95×10^−5^
	GO:0030595	leukocyte chemotaxis	9	2.13×10^−5^
	GO:0023061	signal release	14	2.38×10^−5^
	GO:0097529	myeloid leukocyte migration	9	2.69×10^−5^

Top 10 terms for each category.

### 3.7 Combined metabolomics and transcriptomics analysis

**Figure 7:**
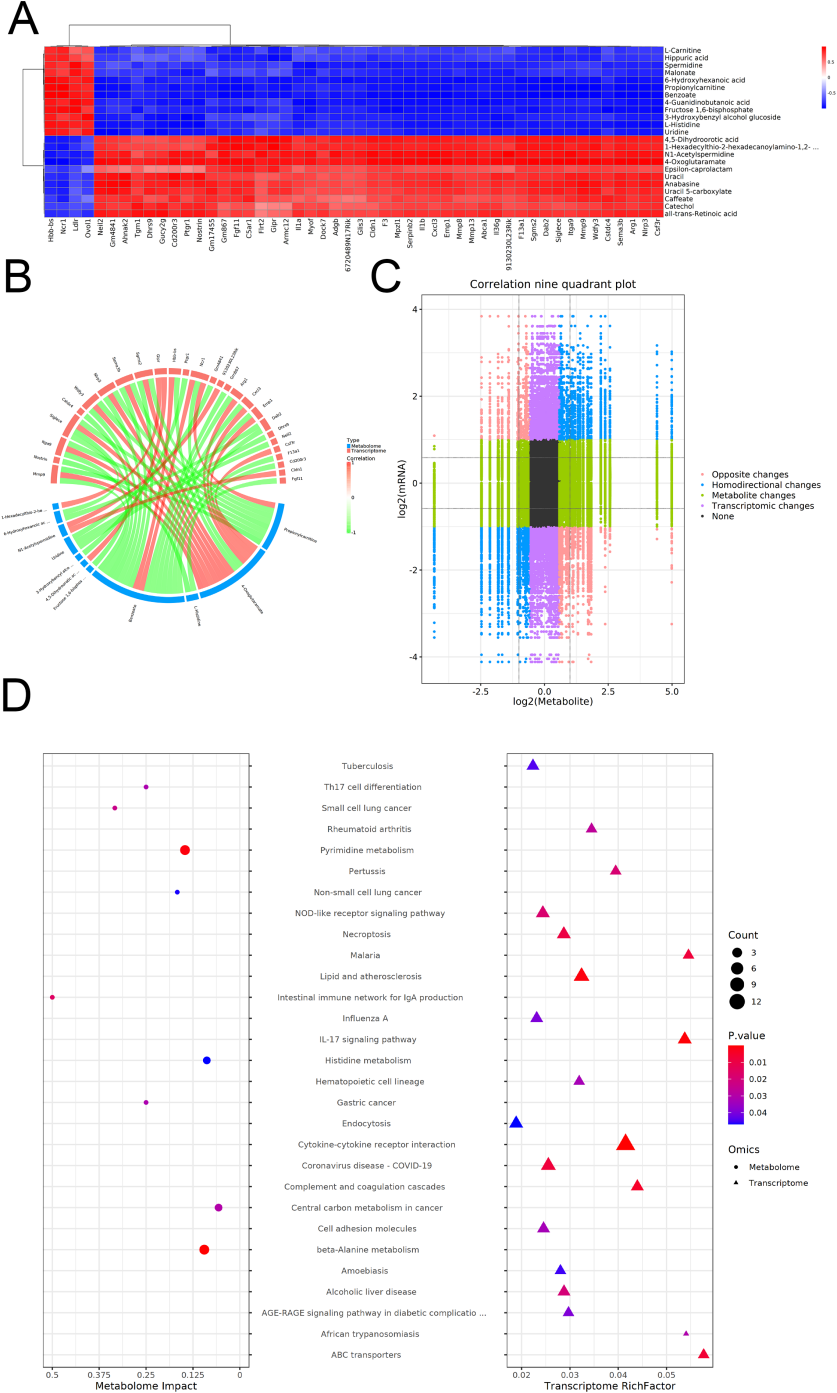
Co-analysis of metabolomics and transcriptomics after co-incubated of EgCF with mouse splenic lymphocytes. **(A)** Heat map of correlation clustering, where redder colors indicate stronger positive correlations and bluer colors indicate stronger negative correlations. **(B)** The correlation chord diagram shows red nodes in the upper half representing differential mRNAs, blue nodes in the lower half representing differential metabolites, and lines connecting the nodes indicating the correlation relationship between the two. Red connecting lines represent positive correlation, while green connecting lines represent negative correlation. **(C)** A correlation nine-quadrant plot displays different trends in metabolite and mRNA changes: red dots (Opposite changes) indicate opposite trends, blue dots (Homodirectional changes) show consistent trends, green dots (Metabolic changes) represent metabolic differences only, purple dots (Transcriptomic changes) indicate mRNA differences only, and black dots (None) show no differences in both metabolism and mRNA. Transcriptomic changes indicate mRNA differences only, and black dots indicate no differences in both metabolism and mRNA. **(D)** KEGG enrichment bubble diagram shows the size of the P-value, with redder colors indicating stronger enrichment. The size of the dots represents the number of differential mRNAs or differential metabolites. Circles on the left indicate the metabolome, while triangles on the right indicate the transcriptome. If a pathway corresponds to having both triangles and circles, it means that the pathway is enriched in both histologies.

To elucidate the integrated metabolic and transcriptional mechanisms underlying EgCF-induced immune modulation, we performed a multi-omics correlation analysis. Integrated multi-omics analysis revealed significant correlations between metabolites and genes (|correlation| > 0.8,*p*< 0.05).Identify and select the correlation results between differentially expressed mRNAs and metabolites, then generate a correlation clustering heatmap. For the heatmap display (**Figure 7A**), prioritize and choose the top 50 metabolites and mRNAs, ranked by their P-values from the lowest to the highest. Differential mRNAs and differential metabolites with |correlation| > 0.8 and correlation test p-value < 0.05 were sorted. The top 50 correlations were selected to create a correlation and chord diagram (**Figure 7B**). Pearson correlation calculations were performed for mRNAs and metabolites. A nine-quadrant plot was used to display the distribution of the multiplicity of differences for substances in each subgroup with |correlation| > 0.8 and a correlation test P-value < 0.05, respectively (**Figure 7C**). KEGG enrichment analysis showed 20 pathways enriched in the transcriptome (e.g., IL-17 signaling) and 9 in the metabolome (e.g., Pyrimidine metabolism), with no shared pathways (**Figure 7D**). Despite this, IL-17 signaling (transcriptome) and Pyrimidine metabolism (metabolome) both regulate Th9/Th17 balance:*Hgf* in IL-17 signaling may activate hepatocyte regeneration to counter parasite damage, while pyrimidine metabolism provides nucleotide precursors for lymphocyte proliferation, supporting EgCF-induced Th9 expansion.

## 4 Discussion

EgCF is a complex fluid with various antigens and worm secretions (38). It has cytotoxic effects on lymphocytes (9), but no significant impact on human hepatocyte activity (39). This study aimed to investigate the effects of EgCF on the immune response of mouse splenic lymphocytes and its potential role in parasite establishment within the host. PCNA is an indicator used to evaluate the proliferative status of cells (40). Cyclin D1 is a key regulator of the cell cycle. Our data indicate that the cell groups treated with EgCF exhibit increased proliferation and apoptosis, suggesting that EgCF has a complex regulatory effect on cell fate. On one hand, EgCF may promote the progression of the cell cycle, thereby enhancing cell proliferation. On the other hand, EgCF may also activate apoptotic pathways in certain cells, potentially serving as a compensatory regulatory mechanism in response to the stress associated with increased proliferation. This dual effect may be influenced by the dosage of EgCF, the type of cell, or the physiological state of the cell.

Flow cytometry analysis indicated that EgCF enhanced CD3^+^T cell expression and suppressed CD4^+^T cell expression. Early infection with *E. granulosus* activates innate immunity (41), but as the metacestode forms, the CD4^+^T cell-mediated immune response diminishes, allowing *E. granulosus* to grow. Flow cytometry assay suggested that EgCF promoted IL-9 cell expression. Increased IL-9 expression was found in liver tissues of patients infected with *E. granulosus* and *Echinococcus multilocularis* compared to the healthy population (42). When initial CD4^+^T cells are induced by both TGF-β and IL-4, they can differentiate into a large number of IL-9-secreting Th9 cells (43). TGF-β and IL-4 influence Th9 differentiation by activating PU.1 and IRF4 expression. Correlation analysis showed that the increase in TGF-β, IL-4, and IL-9 was positively correlated with the EgCF concentration. In a mouse gastric cancer model, increased TGF-β and IL-4 promoted IL-9 secretion by Th9 cells, inhibiting tumor-associated inflammation and metastasis (44). In a melanoma model, long-term antibiotic treatment disrupted intestinal flora, reduced IL-4 and TGF-β expression, and decreased IL-9-producing T cells in the tumor microenvironment (45). Additionally, Haemonchus contortus proteins were found to promote Th9 differentiation and IL-9 expression (46, 47).

PU.1 is a crucial transcription factor for IL-9 secretion by Th9 cells. IL-9 expression is compromised in the absence of PU.1 in mouse CD4+ T cells, while its upregulation boosts IL-9 production. IRF4 is required for Th9 cell development, and its absence prevents IL-9 secretion in Th9 cells. In experiments, the expression of PU.1 and IRF4 increased with rising EgCF concentration, and their elevation was positively correlated with IL-9 production. This supports the role of PU.1 and IRF4 in IL-9 secretion by Th9 cells. In osteotriol-mediated regulation of Th9 cell differentiation, downregulation of PU.1 and IRF4 reduced IL-9 secretion (48). Overexpression of Foxp2 in an asthma model inhibited Th9 differentiation and reduced IRF4 and BATF expression (49). In colorectal cancer patients, Th9 cells and IL-9 promoted tumor growth, with both IL-9 and PU.1 elevated in intestinal mucosal tissues, and tumor growth inhibited in IL-9 or PU.1 knockout mouse models (50).

To understand the changes in the metabolic levels of splenic lymphocytes in mice affected by EgCF, metabolomics was used to detect and analyze the metabolites of splenic lymphocytes after intervention. A total of 221 secondary differential metabolites were screened, including 12 up-regulated (e.g., Spermidine, Benzoate) and 11 down-regulated (e.g., all-trans-Retinoic acid, Catechol) metabolites. KEGG enrichment analysis identified 24 metabolic pathways, including the beta-Alanine metabolism pathway, with 32 metabolites, 4 of which were differential, including 3 up-regulated and 1 down-regulated metabolite. Oral administration of alpha-difluoromethylornithine did not significantly affect *E. granulosus* development or cause depletion of polyamines in cysts (51). *In vitro* inhibition of *Toxoplasma gondii* by Estradiol Benzoate and Octyl Gallate (52). All-trans-retinoic acid promotes the differentiation of myeloid-derived suppressor cells into mature cells, inducing E. granulosus to overexpress Ly6G and Ly6C (53). Transcriptomic analysis of the intervened splenic lymphocytes revealed 141 differentially expressed genes, with 124 up-regulated (e.g., Hgf, Myof) and 17 down-regulated (e.g., Ncr1, Ldlr). GO analysis showed significant enrichment in extracellular space (cellular component), receptor ligand activity (molecular function), and neutrophil migration (biological process). KEGG enrichment analysis revealed significant enrichment in the Necroptosis, Notch signaling, TGF-β signaling, Lipid and atherosclerosis, Purine metabolism, and IL-17 signaling pathways. Multi-omics analysis combined transcriptomics and metabolomics to explore biological processes, filtering key genes, metabolites, and pathways. Through correlation analysis, 4-Oxoglutaramate, Benzoate, and Propionylcarnitine were identified as frequently associated compounds. Significant enrichment of spermidine in beta-Alanine metabolism aligns with its role in suppressing macrophage TRAF6 signaling, which may explain EgCF-mediated immunosuppression, while Hgf overexpression indicates activation of hepatocyte growth pathways, possibly compensating for parasite-induced damage.

In this study, various concentrations of EgCF were co-incubated with mouse splenic lymphocytes *in vitro*. It was observed that EgCF could enhance the proliferation and apoptosis of mouse splenic lymphocytes. Simultaneously, it was noted that EgCF could modulate the host’s immune response by up-regulating Th9 cell-associated cytokines such as IL-4, TGF-β, and IL-9, along with related transcription factors PU.1 and IRF4. Through the application of metabolomics and transcriptomics, researchers can identify potential metabolic pathways or key transcription factors. This can enhance our understanding of the immunopathological mechanisms of the disease and offer a new theoretical foundation for disease prevention and treatment. This may help to reveal the pathogenesis of echinococcosis and the regulatory mechanisms of the host immune response, providing new ideas and methods for the prevention and treatment of the disease. Although transcriptome-metabolome integration revealed no co-enriched KEGG pathways, IL-17 signaling (transcriptome) and pyrimidine metabolism (metabolome) both contribute to Th17/Th9 balance, implying a novel axis for future therapeutic targeting.

## Data Availability

The data presented in the study are deposited in the OMIX, China National Center for Bioinformation / Beijing Institute of Genomics, Chinese Academy of Sciences (https://ngdc.cncb.ac.cn/omix: accession no.OMIX011434).
